# Has the social justice approach become pervasive as a tool for fighting HIV in women? The case of Zambia

**DOI:** 10.1186/s13104-019-4420-z

**Published:** 2019-07-03

**Authors:** Choolwe Muzyamba

**Affiliations:** 1 Maastricht Graduate School of Governance/UNU-Merit, Maastricht University, Maastricht, The Netherlands; 2 A9 Marshlands Village, Box 32379, Lusaka, Zambia

**Keywords:** Social justice, Women, HIV/AIDS

## Abstract

**Objective:**

Research has constantly shown how gender-based social inequality in countries like Zambia leads to disproportionately higher HIV prevalence rates among women aged 15 to 45 years old. As a response to this, the social justice approach in HIV response has become gold standard. Despite its continued application, little is known about how this approach is received and experienced by the people it is meant to serve. Thus the aim of this study is to fill this gap by investigating Zambian women’s interpretation and experience with the social justice approach as a tool for fighting HIV infection.

**Results:**

The social justice movement’s role in highlighting different gender-based social inequalities was praised by our participants; however, there are several ways its application proved counterproductive in the context of Zambia. Thus, in many ways our respondents remained repugnant to the approach thereby closing down opportunities for fighting social inequality and HIV. Overall, our findings indicate that rather than definitively establishing the social justice approach as an incontestable good, there is more to benefit from paying attention to the diverse ways it is viewed by people it is meant to serve.

## Introduction

In Zambia, the HIV prevalence rate for women is around 16%, which is 25% higher than that of men [1]. Other than biological factors, research has consistently shown that this difference is caused by complex gender inequalities which filter women’s agency to determine safer sexual practices [2]. Thus in order to ameliorate the HIV scourge, several public health programs have resorted to adopting the social justice approach. The social justice approach involves the designing and implementation of HIV/AIDS programs based on social justice principles centered around the promotion of rights of women [3]. This approach has been popularized and adopted by funders, local Non-Governmental Organizations(NGOs) and the Zambian government to serve as an antidote to the HIV scourge [4]. Despite the continued application of the social justice approach, little is known about how effective it is in inspiring behavioral change among its targets, the Zambian women. It also remains unclear to what extent it is appropriate in tackling the social inequality responsible for high HIV rates among women in Zambia. Thus the aim of this study is to fill this gap by investigating Zambian women’s experiences with the social justice approach as a tool for fighting HIV infection.

### Theoretical framework

The study is located against the background of Social Representations Theory (SRT). The SRT holds that people in society give meaning to a new or strange social phenomenon based on shared experiences and as a result of social interaction with others [5]. Thus in order for one to assess the usefulness of interventions, there is need to investigate the social representations of that particular phenomenon in the given society. The Social Representation Theory further postulates that in order to understand the impact, experience and acceptance of a given social phenomenon (in our case the social justice approach), it is important to investigate local people’s interpretation and characterization of this phenomenon [5]. We thus use this theory heuristically to guide our investigation, interpretation and presentation of findings. In this sense, the framework allows us to investigate in what ways local women find the social justice approach useful including its limitations.

## Main text

### Methods

#### Ethical clearance

Before the study was conducted, we got written ethical clearance from the National Health Research Authority of Zambia (NHRAZ). We also collected written informed consent from participants.

#### Setting

The study was conducted in Lusaka city; Lusaka is the political and economic capital of Zambia. Lusaka has a population of around 1.7 million people and also retains some of the highest HIV rates in the country. The city also has the majority of NGOs conducting social-justice centered HIV prevention activities. It is for this reason that Lusaka was selected as a case study.

#### Sampling

Participants were recruited through convenient and purposive sampling techniques. In total, 63 women were selected to take part in the study. All participants were beneficiaries of social-justice related HIV prevention programs in communities; this was through taking part in the ‘rights-for-women’ advocacy program which was centered around promoting women empowerment and rights of women to fight HIV. The selected participants varied in marital status, age, occupation and educational level. Variety of participants was meant to increase diversity of opinions expressed.

#### Data collection

In order to collect data, 6 different FGDs were held separately in different parts of Lusaka (Misisi, Kanayama, Matero, Kabwata, Mtendere, Chilenje). Our FGDs consisted of about 10 or 11 participants. FGDs were conducted in Chinyanja the local language and English was used were possible. An FGD guide was used to guide the discussion. This guide included about 12 general questions ranging from reasons why participants participated in the HIV prevention programs, what their experience was, what they think was good or bad about the programs, what the value of the programs was in the fight against HIV, what was lacking in the programs etc. We also asked follow up questions to ensure thorough discussions.

#### Analysis

Thematic analysis technique with the help of NVivo software was used to analyze the data. Thematic analysis is a technique used to examine and describe different phenomenon by relying on emerging themes arising from the data [6]. Here, similar opinions from participants were clustered together to build global themes. The summary of these results arising from the data analysis are presented in Table 1.

**Table 1:** Summary of qualitative results

Global theme	
Positive characterization	Important in highlighting discrimination Made women aware of their rights Opens up conversation on social inequality Provides women with information on where to report abuse Opportunities to gain information on HIV and sexual rights and health
Negative characterization	Lack of flexibility in implementation Conflict with cultural and religious values Failure to address women’s priorities and day-to-day realities Social justice is an “un-Zambian” Western concept Disrupts survival networks (marriage) of unemployed women Women felt disconnected with the approach Implementation of approach done in a way that is too confrontational It conflicts traditional cultural and religious values Alienates potential allies Creates enemies Ignores men in the conversation It is merely a symbolic gesture without substantial economic survival opportunities Champions of the approach are out of touch with local realities Ignores local strategies It is neocolonial It is a hegemonic imposition of western culture and standards

### Results

By use of the Social Representation Theory, our results showed that although representations of the social-justice approach varied, respondents in general noted that an emphasis on social-justice, in particular, rights of women and female empowerment was necessary for addressing the complex drivers of the HIV epidemic in Zambia. They argued that the social justice campaign proved effective in raising awareness about discrimination and inequality that women suffer:

*“It is clear that these campaigns have brought to light many issues such as social inequality that most women face and how this then pre*-*disposes them to the risk of HIV.”*

It was clear that the majority of the respondents appreciated the “spirit” behind the social justice movement but remained critical of the manner it was being applied in Zambia. Firstly, they highlighted that the confrontational nature of the approach (in which women were expected to challenge their male counterparts) was consistently alienating potential male allies. Our participants noted that the social justice tactics and conversations premised on ‘calling-out’ perpetrators gave birth to ‘enemies’ instead of ‘allies’. This therefore made it difficult for the well-intended message of curbing social inequality to actualize within communities.

*“It is a game of calling out each other. It has been packaged as a confrontational initiative which gives no room to building workable relationships with male counterparts. Other women also see this as problematic.”*

In the fight against HIV, our participants also questioned what they termed as a misrepresentation of perpetual outrage against men as a proxy for women empowerment. They postulated that social justice campaigns had become “toxic” in that they were focused on aggressively-demanding change from men instead of inspiring collaboration. They also wondered why men who were branded as perpetrators were left out of the conversation. They therefore argued that such limitations made it difficult for them to embrace the approach.

*“Where is the logic in being angry? It is really toxic these days. It’s all about challenging men, while at the same time leaving the very perpetrators (men) out of the conversation.”*

Further, the participants were concerned about who controlled the ‘language’ of social justice in relation to HIV. Their worry was expressed in two ways: a) the language and narrative around social justice was controlled by women in position of power (such as high class NGO workers). b) the narrative on social justice was riddled with neocolonial tyrannical tendencies as seen from its conceptualization and implementation which reflected a western characterization. The combination of the above led to a detachment of the social justice movement from the lived experiences of local women. Local women thus felt that this state of affairs contributed to the false idea that there was only one way to think about, talk about, and ultimately do activism on social justice. This further closed down any possibilities of incorporating a plurality of tactics in dealing with social inequality thereby leaving citizens (both male and female) repugnant to social justice campaigns much to the detriment of the HIV response.

*“Who is in control? Us? This is a movement of and by rich women who are driving fancy cars. They have money; they do not know our situation. They speak from air*-*conditioned offices after receiving funding from the West. This is why people are not taking them serious these days.”*

Moreover, the vast majority of the discussion focused on the inappropriateness of the social justice approach which was accused of being incompatible with cultural and religious values of Zambia due to its emphasis on challenging traditional authority hierarchies such male-dominance in marriages. This was out of touch with our participants’ realities because the approach specifically threatened relationships which they relied upon for their sustenance. They further noted that the social justice campaigns were merely symbolic gestures that did not guarantee tangible alternatives for economic survival for women who in most cases were unemployed.

*“We have our own culture; this is the same culture that has allowed us to live in peace in marriage; what is the value of adopting this western*-*precipitated culture that threatens our very survival. Men provide for women here.”*

### Discussion

The Social Representation Theory helped illuminate the varied experiences of the social justice movement from the perspective of the women it is meant to serve. In this sense, our results indicate that the social justice approach is seen as useful in highlighting the gender-based social inequalities which are responsible for the disproportionately higher HIV rates in women. The approach also seeks to tackle the symbolic drivers of HIV which are rooted in religious and cultural systems of discrimination against women. Yet at the same time, there are several ways in which this approach contradicts many of our participants’ priorities, needs, and their version of effective-implementation.

That notwithstanding, our study highlights a problematic bourgeois—precipitated sociocultural imposition of hegemonic understandings of social justice on ‘less-powerful’ women without regard to their realties. A western understanding of social justice which is usually channeled through affluent local women has failed to find fertile ground among the most vulnerable women in communities [7]. The approach has been consumed with a false assumption that there is only one way to think about, talk about, and ultimately promote social justice [8]. In Zambia, where most women are uneducated and unskilled, the ‘modern’ worldviews associated with the social justice movement represents a world that these local women don’t see themselves as having access to. It is for this reason that this approach as a tool to combat HIV has failed to achieve local buy-in.

Further, in line with sentiments by Farmer [8] and Englund [9], our study illustrates local women’s frustration with HIV-prevention programs that advocate for awareness of social justice while neglecting issues like poverty, unemployment, religion and culture. Among most Zambian women, cultural and religious understandings of gender relations serve as important economic and social protection mechanisms. Although social justice narratives claim to address deep-rooted social inequalities, in reality they do so only symbolically without providing tangible social and economic alternatives to local women. In settings where men control access to economic and social resources- including privileged access to scarce job opportunities, women who are mostly unskilled rely on the support of their male counterparts [10, 11]. Just as other studies [7] from Zambia have shown, without providing alternatives, the social justice approach could be harming the people it is meant to serve. Its insistence on aggressively challenging socially unequal gender relations while ignoring the role these relations play in the survival of women is seen as counterproductive.

Further, in agreement with another study from Zambia [7], the social justice approach in Zambia is viewed as a neo-colonial project which seeks to disrupt cultural and religious networks of survival. Many of our participants consequentially called for locally-informed approaches to HIV prevention initiatives. The ones which are alive to the lived realities of local women. Our respondents also suggested that rather than branding men as the “enemy to be fought”, involving them as partners in the fight against HIV had the potential of yielding better outcomes. Thus instead of seeking for perfection, our participants seemed to favor a social justice of imperfection and responsibility; one which constantly investigates its own reproduction of and complicity in sustaining social inequality. This type of social justice intervention should be based on seeking context-specific strategies and plurality of tactics in bringing about a social change that is more feasible in the given context.

## Limitations

Findings from this study are based only on views of respondents who were located in only one of the ten provinces of Zambia. This therefore may have limited the variety of experiences with the social justice approach vis-à-vis HIV response. However, we posit that this study from Zambia is adequate and relevant in highlighting insights into ways the social justice approach creates or inhibits opportunities for HIV response in Zambia

## Data Availability

The data generated and/or analyzed during the current study are not publicly available due to the fact that they contain people’s private information including their HIV status but are available from the corresponding author on reasonable request.

## Abbreviations

FGD: Focused Group Discussion
HIV: human immunodeficiency virus
NGO: Non-Governmental Organization
SSA: Sub-Saharan Africa
WHO: World health Organization
